# 

**Published:** 1881-10

**Authors:** 


CONTENTS.

| | PAGE. |
|---|---|
| Nature's Methods | 363 |
| Auscultation | 365 |
| My Ship at Sea (Poetry) | 367 |
| The Best Part of Man's Life | 367 |
| A Monster of the Sea | 368 |
| Eyebrows | 371 |
| Benefits Derived from Washing in Cold Water | 372 |
| A Habit of Sleep | 373 |
| A Cure by Imagination | 374 |
| Scarlet Fever | 375 |
| Grow Beautiful | 378 |
| Aims of the Journal | 379 |
| Tea and Coffee | 380 |
| Milk | 381 |
| C)rns Cured | 382 |
| Going to the South | 382 |
| The Nature of Cough | 384 |
| Physical Education of Children | 385 |
| How to Keep Cool | 387 |
| The First Sign of Consumption | 387 |
| Summer Complaint | 388 |
| Ventilation | 389 |
| D n't Drown with a Shingle within Reach | 391 |
| Advantages of Sunlight | 392 |
| Punishment by the Cangue in China | 393 |
| The Moon and the Weather | 393 |

